# Self-Medication in the COVID-19 Pandemic: Survival of the Fittest

**DOI:** 10.1017/dmp.2021.173

**Published:** 2021-06-08

**Authors:** Kiran Rafiq, Shagufta Nesar, Humaira Anser, Qurat-ul-Ain Leghari, Alisha Hassan, Alina Rizvi, Aleeza Raza, Zafar Saied Saify

**Affiliations:** 1 Institute of Pharmaceutical Sciences, Jinnah Sindh Medical University, Karachi, Pakistan; 2 Jinnah College of Pharmacy, Sohail University, Karachi, Pakistan; 3 Zia-ud-Din College of Pharmacy, Zia-ud-Din Medical University, Karachi, Pakistan; 4 International Center for Chemical Sciences, H. E. J. Research Institute of Chemistry, University of Karachi, Karachi, Pakistan

**Keywords:** COVID-19, coronavirus, self-medication, Zenodo scale survival of fittest, pandemic

## Abstract

**Objective:**

After the World Health Organization (WHO) declared coronavirus disease 2019 (COVID-19) a pandemic, intense efforts to combat the novel coronavirus were undertaken, with many fatalities in most regions of the world. The high fatality rate and socioeconomic collapse affected the health of uninfected individuals because healthcare measures and scheduled clinical and hospital visits were avoided by people in an attempt to reduce their exposure to the contagion. The general population began self-medication practices as means to safeguard against exposure to the virus.

**Methods:**

The present study investigated the effectiveness of self-medication compliance among the general population. For this purpose, a questionnaire on the Zenodo scale was developed and adults and teen respondents were asked to complete it, after providing consent. The data gathered were analyzed using IBM SPSS Statistics Version 26.

**Results:**

The study amazingly found high compliance with self-medication among the focused population during the period of COVID-19. Estimated results showed a highly significant correlation of 0.000, *P* < 0.05, between the adaptation of self-medication and pandemic situation, which was estimated from chi-squared and Fisher test results.

**Conclusions:**

However, the fear of coronavirus made the practice, or malpractice, a survival of the fittest, innate ability of human nature.

The highly contagious and serious nature of novel coronavirus disease 2019 (COVID-19) led to exponential numbers of positive cases day after day in almost all the regions of the world. Ultimately, the World Health Organization (WHO) declared an epidemiologic emergency that developed into a global pandemic, leading to a huge socioeconomic crisis. The rapidly deteriorating condition complicated already overwhelmed healthcare systems globally, and more specifically in third-world countries where hunger is a more demanding issue than health.^1^ The limited resources of and continuous lockdown situations in some afflicted countries destroyed the backbone of their economies, especially those countries that had made great strides in improving and upgrading their healthcare systems.^2^

## The General Population

Social distancing, quarantine, and self-isolation measures were established to keep people safe from the virus. The efforts worked in the countries that followed these guidelines strictly; the spread of the virus slowed. Along with messages from healthcare practitioners and governing bodies, other strategies, such as electronic media, SMS services, mask distributing campaigns, and WhatsApp messaging were effective in making people aware of facts about the disease and its prevention as well.^3^ Nevertheless, the parallel and comorbid fear of the virus provoked anxiety and stress that made people anxious and nervous; also, the uncertainty of what could happen was overwhelming and emotionally intolerable. Public health actions to reduce the spread of COVID-19, such as social distancing, made people feel lonesome and inaccessible. Under normal conditions, social gatherings help individuals cope with stress by providing a sense of belonging and connection, which strengthens the community.^4^

The COVID-19 virus is highly contagious. Entire families and circles of friends have died after exposure to 1 infected individual.^5^ Persons with chronic health conditions are especially susceptible to the serious effects of the disease.

Worsening of mental health conditions can lead to the development of posttraumatic stress disorder (PTSD), a disorder that usually is attributable to exposure to remarkably intimidating or horrific events. Pandemics can account for the development of PTSD symptoms among the general population and healthcare practitioners; optimistic and encouraging coping approaches can help.^6–8^ An investigation, conducted in China during the current COVID-19 outbreak, studied the factors associated with posttraumatic stress among nurses exposed to COVID-19 and confirmed the fragility of psychological stability, which could be strengthened by counseling and other mental health therapies.^9^ Another study, carried out among the Italian population regarding COVID-19 PTSD, found that stress symptoms were observed with high frequency.^10^ Psychological intercession to diminish mental health decline was deemed essential during the COVID-19 pandemic. These findings confirmed that the current COVID-19 pandemic caused fear, which led to anxiety and stress that influenced normal human social and trade activities.

In addition to social and trade activity disruption, academia was highly affected also; perhaps the most important detrimental impact of the pandemic though was observed on healthcare practices.^11^ People were acutely aware of COVID-19’s highly contagious nature and transmission of the pathogen through direct expulsion of droplets by means of coughing, sneezing, and vocalizing. People then became more apprehensive and feared becoming a victim as reports emerged substantiating the persistence for several hours of infectious moiety on nonliving surfaces.^12^ The possibility of viruses remaining viable in aerosols leads to airborne transmission when people are close together. Certainly, reasonable verification exists about the airborne transmission of coronavirus, and diffusion in closed environments may occur even up to 10-meter distances from the secretion source.^13,14^ In Wuhan, air samples collected from different hospitals showed the presence of the fatal virus in the air and even in surrounding areas, enough to confirm the airborne means as an important way for dispersion.^15^

All the facts regarding exposure to the virus, such as public distancing and transmission through the air, negatively impacted routine health-care practices in communities, such as clinic visits for chronic and acute ailments, scheduled consultations, vaccinations, regular check-ups by physicians. Almost all such activities were prohibited during the pandemic. The COVID-19 situation encouraged “self-medication,” the practice of treating yourself or your family without consultation from any healthcare practitioner.^16^

The phenomenon of self-medication was widely practiced and highly variable in different communities according to gender and age. Many studies have been conducted on the issue and established that the medical community frowned on the practice and offered suggestions and promotions to improve outcomes, such as awareness campaigns, medical camps, low-cost consultancy, and the use of other healthcare facilities.^11^ Unfortunately, most of these options do not fulfill the standard operating procedures and criteria required for the current pandemic situation to prohibit exposure to coronavirus. All above-mentioned activities vanished out of the guidelines for violating isolation and social distancing mandates. Because clinic consultations and visits will likely expose one to the virus on a surface or in the air, or to someone who is a carrier, the likelihood of becoming infected is higher that if one stays at home. Accordingly, the population began practicing self-medication as a survival of the fittest measure to stay safe and avoid getting exposed to a potentially life-threatening or fatal coronavirus pathogen.^17^

The present study was designed to analyze self-medication practices among the general population, that is, instead of becoming badly ill by being exposed to COVID-19 while seeking health care, perhaps it is better to self-administer and self-manage health conditions. However, the consumption of medicines without a prescription or doctor’s opinion carries risks. Most people do have easy access to and availability of drugs with or without a prescription, and they adopted self-care during the pandemic.^17,18^

The cross-sectional design aimed to investigate the factors and motives involved in self-medication among the general population during the COVID-19 pandemic. The objectives of the study included evaluation of the pervasiveness of self-medication during specific outbreak periods according to different sociodemographic dimensions, as well as the occurrence and possibility of side effects, preventive measures, and available resources. It was hypothesized that the study outcomes would establish that self-medication was not an excellent, but rather a supportive and significant, tool for self-care and well-being during the highly contagious COVID-19 pandemic.

## Methods

### Study Design

The present large study was conducted in Pakistan during the COVID-19 pandemic. The study population consisted of 920 inhabitants that practiced self-health management for general diseases throughout the pandemic. An online 13-point questionnaire was developed on the Zenodo scale and circulated among different groups of the general population by means of Whatsapp, Messenger, and e-mail until the required sample size was achieved. The shared questionnaire was kept anonymous to ensure confidentiality and trustworthiness. The study period was from March to August 2020.

### Data Anthology

The questionnaires developed included questions about the sociodemographic characteristics of respondents, such as age, gender, and profession. The required questions were focused on the frequency and likelihood of adaptation intended for self-medication during the outbreak. Other queries were about having any chronic disease, possible side effects due to self-medication, and the method of monitoring disease activity.

### Data Analysis

The information and data collected were encapsulated using descriptive and inferential statistics that were portrayed through tables, graphs, percentages, and cross-tabulation. Further collected data were scrutinized by means of the Statistical tool, IBM SPSS Statistics Version 26. Pearson chi-square and Fisher exact test were used to determining factors affecting an individual’s desire to opt to self-medicate during the pandemic. Associations with *P* < 0.05 were deemed significant.

### Inclusion and Exclusion Criteria

The study was designed to focus on adult and teenage individuals of both genders; children and the elderly were exempted. Furthermore, pregnant women were not included in the study as well.

## Results

### Sociodemographic Values of the Study

The study was targeted to collect 1000 respondents, but approximately 92% (*n* = 920) questionnaires were responded to. The survey was answered by members of the general population: females (*n* = 740) 80.4%, males (*n* = 180) 19.6%, adults (*n* = 754) 81.9%, teenagers (*n* = 166) 18.0%, professionally employed (*n* = 123) 13.3%, housewives (*n* = 222) 24.1%, self-employed (*n* = 42) 4.5%, and students (*n* = 533) 57.9% (Table 1).

**Table 1.** Sociodemographic characteristics of respondents during the COVID-19 outbreak

		***N***	(%)
Gender	Female	740	80.4
	Male	180	19.6
Age	Adult	754	81.9
	Teenaged	166	18.0
Profession	Employed	123	13.3
	Housewife	222	24.1
	Self-employed	42	4.5
	Students	533	57.9

### Prevalence and Reported Reasons for Self-Medication

The study outcomes showed the frequency of falling ill once (*n* = 394), 62.9%, and twice 19.1% (*n* = 120) more prominently during the COVID pandemic. Of interest, among the overall population, a total of (*n* = 684) 74.3% of people avoided and refused the doctor visit and 25.7% (*n* = 236) opted for the choice. Among adults, 754 (*n* = 508) 67.3% adopted self-medication, whereas among teenagers, 166 (*n* = 78) 46.9% were found to be in favor of self-medication. Regarding profession, students (*n* = 355) then housewives (*n* = 126) were more in favor of self-management. According to the data among those who fell ill once (*n* = 626) in COVID-19 (*n* = 394), 62.9% did not visit for consultancy and availed themselves of self-medication; even those who fell ill twice *n* = 168, (*n* = 120) 71.4%, treated themselves through self-medication. The respondents were also solicited concerning the conditions that imposed self-medication. In addition to suffering from conditions such as skin allergy, rhinitis, diarrhea, and others, the most prominent symptoms appeared to be flu/cough in *n* = 395, and among them (*n* = 281) 71.1% preferred self-treatment, whereas *n* = 238 caught a fever during COVID-19, and among them *n* = 150, 63% practiced self-treatment (Table 2).

**Table 2.** Adaptability analysis of self-medication during the COVID-19 outbreak

	Pearson chi-squared value	Asymptotic significance	Fisher exact test
Age group	24.451^a^	0.000	0.000
Profession	8.052^a^	0.045	NA
Number of times you fell ill during COVID-19 (March 2020-August 2020)	7.801^a^	0.05	NA
What kind of disease did you suffer from?	33.355^a^	0.000	NA
Did you avoid going to the clinic/hospital due to COVID-19?	67.471^a^	0.000	0.000
Did you have any ailment/disease?	53.406^a^	0.000	NA
Did you avoid or limit your scheduled/routine visits to clinics/hospitals due to COVID-19?	122.125^a^	0.000	0.000
Your opinion for opting for self-medication during COVID-19	180.417^a^	0.000	0.000
If yes, did you feel side effects after self-medication?	69.573	0.000	0.000

a additional data and cross-tabs in Supplementary Online Tables.

### Health Status and Clinic Visit History

In the response to the question about having any disease, approximately (*n* = 514) 55.8% of participants said they did not have diabetes, hypertension, allergy, digestive disorders, or other diseases. However, the positive responses for self-treatment were amassed from the participants who said they have diseases, specifically diabetes, allergy, and digestive disorders, as they even avoided the scheduled and routine consultancy and visits due to the alarming trepidation of infection and maximum exposure to the virus. The data showed a significant relationship between the 2 variables. The chi-squared test was estimated significant (0.000, less than 0.05) as the scheduled visits were ignored by the population, even by those whose health and disease management rely highly on proper and timely advice of a physician or other healthcare professional.

A very strong significant correlation was observed between avoiding a scheduled visit due to the possibility of exposure to coronavirus and adoption of self-medication instead of visiting for consultancy, with *P*-value obtained 0.000 (less than 0.05) from both Pearson chi-square and Fisher exact test. According to the analysis, a very deep correlation linking regular, frequent, required consultancy, visit, and the self-medication (0.000 from Fisher exact test) that enough strong evidence of fear of going to hospital or a clinic visit leading to adaptation of self-medication during COVID-19 more frequently compared with a normal healthy period than before the COVID-19 period (Table 2).

### Impact of Self-Medication Side Effects

The best conclusion of this study was that negligible side effects of this practice during COVID-19 were observed. The outcomes revealed that the practice of self-medication, with its potential to jeopardize the health, was amazingly popular, with 72.1% of the population opting for the practice as safe and helpful; 29.9% considered it risky and harmful. However, the significant value of Fisher’s exact test of 0.00 (less than 0.05) was established while analyzing the correlation between the opinion and experience about self-medication and preference to opt instead for a doctor visit. The occurrence of side effects was 15%, whereas 85% of participants did not feel any kind of unpleasant outcome associated with the practice. The more prominent side effects were drowsiness, dry mouth, stomachache, among a small portion of the population, and 24% of the population stopped the self-management medicine, while others managed the unpleasant effects through different measures, such as home remedies, increased water intake, lowering dose, or other means (Table 2).

## Discussion

The state of affairs creates an impact on human psychology and associated behaviors. The uncertainty, leading to anxiety and apprehension, has been observed in different global outbreaks, such as Ebola, severe acute respiratory syndrome (SARS), and now with a high intensity in COVID-19 as well.^19,20^ The outcome of such threats hinge on the decisions taken and priorities are chosen that, in turn, alter the lifestyle. In the current pandemic, social distancing^21^ and isolation were effective to vary degrees to combat the threatening and alarming circumstances around the globe. Isolation strategies and giving up travel plans proved to be a successful preventive measure to reduce the spread of the pathogen in communities all over the world.^22,23^ The “stay at home” strategy and long-term isolation guidelines impeded the population from visiting hospitals and clinics, which prohibited possible exposure to the coronavirus. Even with the uncertain conditions related to the option of self-medication, healthcare-seeking behavior was altered. The pandemics are equally responsible for encountering negative impacts. Along with socio-economic disturbances, the psychosocial state and mental health of victims, people around the victims, and the non victimized as well have been influenced.^24^ In short, the whole community subjected itself to self-treatment as a reaction to the panic caused by the contagious virus,^22,25^ as the virus has been established as deadly, having no mercy even for pregnant women for whom death may occur and high chances of transference of the pathogen directly to the fetus.^26,27^

All of these facts and figures about the coronavirus made the situation highly terrifying and directed the community to avoid exposure to the virus at any cost. This preventive measure enhanced the decision to adopt self-treatment.^28^ The present study is focused to portray the factors leading to and outcomes of opting for self-medication by the general population in response to the current outbreak of COVID-19.^29,30^

In the present study, a sample size of 920 persons from the general population was studied through a 13-point questionnaire; data were further analyzed through IBM SPSS Statistics Version 26. The majority of the male gender (74.7%) was found to adopt self-medication, specifically adults as compared to teenagers. A very strong association was found between self-medication use and age. Other studies have also proven that elderly persons are more prone to opt for self-medication in regard to over-the-counter drugs and other alternative medicines.^31^ Elderly persons have more confidence to use older prescriptions and to rely on the opinions of friends and family; hence, that confidence worked more in COVID-19 to choose self-medication as a first line of treatment, to avoid in-person professional medical care.^32–34^ A similar pattern was observed among the professionals who have self-employment businesses set up; a significant correlation, estimated from Pearson chi-squared and different factors, was seen for adopting this practice among such a population and drug buying. Purchasing medications were considered less expensive than consultancy fees. Continuing the self-medication practice was observed to be more economical during the economic downfall faced globally during a pandemic because of complete and partial lock down.^35,36^

Ailments and disorders are a fact of normal human life and sometimes interaction with different pathogens boosts immunity.^37,38^ According to the feedback among the target population, the majority fell ill once during the study period. A significant relationship was shown between the self-medication practice and cough and flu symptoms, which are the initial symptoms of COVID-19.^39,40^ Moreover, the majority had no previous history of any disorder, such as diabetes, allergy, digestive problems, and showed positive interest in adopting the self-management practice to avoid hospital and clinic visits. All the scenarios and opinions favored self-medication and were observed to be supported through a strong and positive association (0.000; *P* < 0.05), all of which are the consequences of dread of potential exposure to COVID-19 being more likely from hospitals and interaction with pathogen carriers.^41-^ ^44^

The avoidance of hospital visits was probably also due to the accessibility of opinion from family and friends who are healthcare practitioners, including doctors, nurses, and pharmacists. A total of 67.7% of the population was found to have a facility that can provide treatment to some extent, however, cannot provide proper consultancy of a physician or a doctor. Of interest, the opinion regarding the risk of side effects of self-medication was observed to be very low, and side effects could be managed through different means, such as home remedies and dose monitoring. Few people worried about self-medication causing the general undesired effects of nausea, drowsiness, and dry mouth.^45-47^ Analgesics were the most preferred medicines observed during COVID, but antibiotics, anti-allergy medications, and cough syrups were used as well for self-management. The outcome shows that side effects may be due to the associated psychological factors of taking medicine that was seemed to be decline before the fear of deadly pathogen exposure.

## Conclusions

After discussing and looking into the frequency and factors involved in adopting self-medication practices during the COVID-19 pandemic as a substitute treatment, it has been concluded that, although the practice had not been proved efficacious, nevertheless, it was greatly accepted as a means to evade the pathogen, as the present study reveals the strong significance of all parameters. In the present situation, the population desires to reduce the risk factors of exposure likely to occur. The brain takes away the body from the source of fear and human psychology works to act for other options, such as self-medication, that could ensure “survival of the fittest” in the current pandemic and isolation. The present and upcoming chaotic times demand appropriately constructed online healthcare systems, online clinics, provision of timely, proper medicines, and related materials eliminate perceived barriers and further strengthen healthcare outcomes.

## Supplementary material

For supplementary material accompanying this paper visit http://dx.doi.org/10.1017/dmp.2021.173. click here to view supplementary material
