# Supplementary material for: Self-Medication in the COVID-19 Pandemic: Survival of the Fittest
Source: Disaster Med Public Health Prep. 2021 Jun 8:1–5. doi: 10.1017/dmp.2021.173 (PMC8367859; doi:10.1017/dmp.2021.173)
Supplement: Supplementary file 1 [file S1935789321001737sup001.docx]

# Appendix:

**Age group.**

**Did you prefer self-medication instead of visiting a doctor**

| **Crosstab** | | | | |
| --- | --- | --- | --- | --- |
| Count | | | | |
|  | | 9. Did you prefer self-medication instead of visiting a doctor | | Total |
|  |  | No | Yes |  |
| Age group | Adult | 246 | 508 | 754 |
|  | Teenager | 88 | 78 | 166 |
| Total | | 334 | 586 | 920 |

| **Chi-Square Tests** | | | | | |
| --- | --- | --- | --- | --- | --- |
|  | Value | df | Asymptotic Significance (2-sided) | Exact Sig. (2-sided) | Exact Sig. (1-sided) |
| Pearson Chi-Square | 24.451^a^ | 1 | .000 |  |  |
| Continuity Correction^b^ | 23.577 | 1 | .000 |  |  |
| Likelihood Ratio | 23.653 | 1 | .000 |  |  |
| Fisher's Exact Test |  |  |  | .000 | .000 |
| N of Valid Cases | 920 |  |  |  |  |

**Profession;**

**Did you prefer self-medication instead of visiting a doctor**

| **Crosstab** | | | | |
| --- | --- | --- | --- | --- |
| Count | | | | |
|  | | Did you prefer self-medication instead of visiting a doctor | | Total |
|  |  | No | Yes |  |
| Profession | Employed | 48 | 75 | 123 |
|  | House-wife | 96 | 126 | 222 |
|  | Self-employed (Business) | 12 | 30 | 42 |
|  | Student | 178 | 355 | 533 |
| Total | | 334 | 586 | 920 |

| **Chi-Square Tests** | | | |
| --- | --- | --- | --- |
|  | Value | df | Asymptotic Significance (2-sided) |
| Pearson Chi-Square | 8.052^a^ | 3 | .045 |
| Likelihood Ratio | 8.005 | 3 | .046 |
| N of Valid Cases | 920 |  |  |

**Number of times you fell ill during Covid-19 (March 2020-August 2020**

**( If you preferred self-medication instead of visiting a doctor)**

| **Crosstab** | | | | |
| --- | --- | --- | --- | --- |
| Count | | | | |
|  | | 9. Did you prefer self-medication instead of visiting a doctor | | Total |
|  |  | No | Yes |  |
| Number of times you fall ill during Covid-19 (March 2020-August 2020) | More than twice | 36 | 42 | 78 |
|  | Once | 232 | 394 | 626 |
|  | Twice | 48 | 120 | 168 |
|  | Very frequent | 18 | 30 | 48 |
| Total | | 334 | 586 | 920 |

| **Chi-Square Tests** | | | |
| --- | --- | --- | --- |
|  | Value | df | Asymptotic Significance (2-sided) |
| Pearson Chi-Square | 7.801^a^ | 3 | .050 |
| Likelihood Ratio | 7.861 | 3 | .049 |
| N of Valid Cases | 920 |  |  |

**What kind of disease you suffered from**

**Did you prefer self-medication instead of visiting a doctor**

| **Crosstab** | | | | |
| --- | --- | --- | --- | --- |
| Count | | | | |
|  | | 9. Did you preferred self-medication instead of visiting a doctor | | Total |
|  |  | No | Yes |  |
| 5. What kind of disease you suffered from | Cough/Flu | 114 | 281 | 395 |
|  | Diarrhea | 6 | 24 | 30 |
|  | Fever | 88 | 150 | 238 |
|  | Other | 96 | 89 | 185 |
|  | Rhinitis | 12 | 18 | 30 |
|  | Skin allergy | 18 | 24 | 42 |
| Total | | 334 | 586 | 920 |

| **Chi-Square Tests** | | | |
| --- | --- | --- | --- |
|  | Value | df | Asymptotic Significance (2-sided) |
| Pearson Chi-Square | 33.355^a^ | 5 | .000 |
| Likelihood Ratio | 33.196 | 5 | .000 |
| N of Valid Cases | 920 |  |  |

**Did you avoid going to the clinic/hospital due to Covid-19**

**If you preferred self-medication instead of visiting a doctor**

| **Crosstab** | | | | |
| --- | --- | --- | --- | --- |
| Count | | | | |
|  | | Did you preferred self-medication instead of visiting a doctor | | Total |
|  |  | No | Yes |  |
| 6. Did you avoid going to the clinic/hospital due to Covid-19 | No | 138 | 98 | 236 |
|  | Yes | 196 | 488 | 684 |
| Total | | 334 | 586 | 920 |

| **Chi-Square Tests** | | | | | |
| --- | --- | --- | --- | --- | --- |
|  | Value | df | Asymptotic Significance (2-sided) | Exact Sig. (2-sided) | Exact Sig. (1-sided) |
| Pearson Chi-Square | 67.471^a^ | 1 | .000 |  |  |
| Continuity Correction^b^ | 66.187 | 1 | .000 |  |  |
| Likelihood Ratio | 65.644 | 1 | .000 |  |  |
| Fisher's Exact Test |  |  |  | .000 | .000 |
| N of Valid Cases | 920 |  |  |  |  |
| a. 0 cells (0.0%) have expected count less than 5. The minimum expected count is 85.68. | | | | | |
| b. Computed only for a 2x2 table | | | | | |

**Did you have any ailment/disease such as … (If you preferred self-medication instead of visiting a doctor)**

| **Crosstab** | | | | |
| --- | --- | --- | --- | --- |
| Count | | | | |
|  | | 9. Did you preferred self-medication instead of visiting a doctor | | Total |
|  |  | No | Yes |  |
| 7. Did you have any ailment/disease such as; | Allergy | 42 | 42 | 84 |
|  | Diabetes | 18 | 0 | 18 |
|  | Digestive disorder | 18 | 12 | 30 |
|  | Hypertension | 0 | 6 | 6 |
|  | No, I don’t | 250 | 514 | 764 |
|  | Other | 6 | 12 | 18 |
| Total | | 334 | 586 | 920 |

| **Chi-Square Tests** | | | |
| --- | --- | --- | --- |
|  | Value | df | Asymptotic Significance (2-sided) |
| Pearson Chi-Square | 53.406^a^ | 5 | .000 |
| Likelihood Ratio | 59.735 | 5 | .000 |
| N of Valid Cases | 920 |  |  |
|  | | | |

**Did you avoid or limit your scheduled/routine visits to clinics/hospitals due to Covid-19**

**(If you preferred self-medication instead of visiting a doctor)**

| **Crosstab** | | | | |
| --- | --- | --- | --- | --- |
| Count | | | | |
|  | | Did you preferred self-medication instead of visiting a doctor | | Total |
|  |  | No | Yes |  |
| Did you avoided or limited your scheduled/routine visits to clinics/hospitals due to Covid-19 | No | 114 | 36 | 150 |
|  | Yes | 220 | 550 | 770 |
| Total | | 334 | 586 | 920 |

| **Chi-Square Tests** | | | | | |
| --- | --- | --- | --- | --- | --- |
|  | Value | df | Asymptotic Significance (2-sided) | Exact Sig. (2-sided) | Exact Sig. (1-sided) |
| Pearson Chi-Square | 122.125^a^ | 1 | .000 |  |  |
| Continuity Correction^b^ | 120.083 | 1 | .000 |  |  |
| Likelihood Ratio | 118.815 | 1 | .000 |  |  |
| Fisher's Exact Test |  |  |  | .000 | .000 |
| N of Valid Cases | 920 |  |  |  |  |
| a. 0 cells (0.0%) have expected count less than 5. The minimum expected count is 54.46. | | | | | |
| b. Computed only for a 2x2 table | | | | | |

**Your opinion on self-medication during Covid-19 (If you preferred self-medication instead of visiting a doctor)**

| **Crosstab** | | | | |
| --- | --- | --- | --- | --- |
| Count | | | | |
|  | | 9. Did you prefer self-medication instead of visiting a doctor | | Total |
|  |  | No | Yes |  |
| Your opinion for opting self-medication during Covid-19 | Risky/Harmful | 184 | 79 | 263 |
|  | Safe/Helpful | 150 | 507 | 657 |
| Total | | 334 | 586 | 920 |

| **Chi-Square Tests** | | | | | |
| --- | --- | --- | --- | --- | --- |
|  | Value | df | Asymptotic Significance (2-sided) | Exact Sig. (2-sided) | Exact Sig. (1-sided) |
| Pearson Chi-Square | 180.417^a^ | 1 | .000 |  |  |
| Continuity Correction^b^ | 178.384 | 1 | .000 |  |  |
| Likelihood Ratio | 178.075 | 1 | .000 |  |  |
| Fisher's Exact Test |  |  |  | .000 | .000 |
| N of Valid Cases | 920 |  |  |  |  |
| a. 0 cells (0.0%) have expected count less than 5. The minimum expected count is 95.48. | | | | | |
| b. Computed only for a 2x2 table | | | | | |

**If yes, then which of the following side effects did you feel after self-medication (If you preferred self-medication instead of visiting a doctor)**

| **Crosstab** | | | | |
| --- | --- | --- | --- | --- |
| Count | | | | |
|  | | Did you preferred self-medication instead of visiting a doctor | | Total |
|  |  | No | Yes |  |
| If yes then which of the following side effects did you feel after self-medication |  | 193 | 388 | 581 |
|  | Diarrhea | 1 | 11 | 12 |
|  | Difficulty in breathing | 1 | 11 | 12 |
|  | Drowsiness | 28 | 25 | 53 |
|  | Dry mouth | 24 | 23 | 47 |
|  | I don't have any side effects | 1 | 5 | 6 |
|  | NA | 0 | 6 | 6 |
|  | Nausea/Vomiting | 27 | 19 | 46 |
|  | No | 0 | 6 | 6 |
|  | No any side effects appears. | 6 | 0 | 6 |
|  | No one | 0 | 6 | 6 |
|  | No side effects | 1 | 16 | 17 |
|  | None | 17 | 13 | 30 |
|  | Nothing | 6 | 18 | 24 |
|  | Nothing happened | 1 | 5 | 6 |
|  | Stomach ache | 28 | 34 | 62 |
| Total | | 334 | 586 | 920 |

| **Chi-Square Tests** | | | |
| --- | --- | --- | --- |
|  | Value | df | Asymptotic Significance (2-sided) |
| Pearson Chi-Square | 69.573^a^ | 15 | .000 |
| Likelihood Ratio | 80.361 | 15 | .000 |
| N of Valid Cases | 920 |  |  |
| a. 14 cells (43.8%) have expected count less than 5. The minimum expected count is 2.18. | | | |

**Questionnaire Analysis**

| **1. Gender** | | | | | |
| --- | --- | --- | --- | --- | --- |
|  | | Frequency | Percent | Valid Percent | Cumulative Percent |
| Valid | Female | 740 | 80.4 | 80.4 | 80.4 |
|  | Male | 180 | 19.6 | 19.6 | 100.0 |
|  | Total | 920 | 100.0 | 100.0 |  |

| **2. Age group** | | | | | |
| --- | --- | --- | --- | --- | --- |
|  | | Frequency | Percent | Valid Percent | Cumulative Percent |
| Valid | Adult | 754 | 82.0 | 82.0 | 82.0 |
|  | Teenager | 166 | 18.0 | 18.0 | 100.0 |
|  | Total | 920 | 100.0 | 100.0 |  |

| **3. Profession** | | | | | |
| --- | --- | --- | --- | --- | --- |
|  | | Frequency | Percent | Valid Percent | Cumulative Percent |
| Valid | Employed | 123 | 13.4 | 13.4 | 13.4 |
|  | House-wife | 222 | 24.1 | 24.1 | 37.5 |
|  | Self-employed (Business) | 42 | 4.6 | 4.6 | 42.1 |
|  | Student | 533 | 57.9 | 57.9 | 100.0 |
|  | Total | 920 | 100.0 | 100.0 |  |

| **4. Number of times you fell ill during Covid-19 (March 2020-August 2020)** | | | | | |
| --- | --- | --- | --- | --- | --- |
|  | | Frequency | Percent | Valid Percent | Cumulative Percent |
| Valid | More than twice | 78 | 8.5 | 8.5 | 8.5 |
|  | Once | 626 | 68.0 | 68.0 | 76.5 |
|  | Twice | 168 | 18.3 | 18.3 | 94.8 |
|  | Very frequent | 48 | 5.2 | 5.2 | 100.0 |
|  | Total | 920 | 100.0 | 100.0 |  |

| **5. What kind of disease you suffered from** | | | | | |
| --- | --- | --- | --- | --- | --- |
|  | | Frequency | Percent | Valid Percent | Cumulative Percent |
| Valid | Cough/Flu | 395 | 42.9 | 42.9 | 42.9 |
|  | Diarhhea | 30 | 3.3 | 3.3 | 46.2 |
|  | Fever | 238 | 25.9 | 25.9 | 72.1 |
|  | Other | 185 | 20.1 | 20.1 | 92.2 |
|  | Rihinitis | 30 | 3.3 | 3.3 | 95.4 |
|  | Skin allergy | 42 | 4.6 | 4.6 | 100.0 |
|  | Total | 920 | 100.0 | 100.0 |  |

| **6. Did you avoid going to the clinic/hospital due to Covid-19** | | | | | |
| --- | --- | --- | --- | --- | --- |
|  | | Frequency | Percent | Valid Percent | Cumulative Percent |
| Valid | No | 236 | 25.7 | 25.7 | 25.7 |
|  | Yes | 684 | 74.3 | 74.3 | 100.0 |
|  | Total | 920 | 100.0 | 100.0 |  |

| **7. Did you have any ailment/disease such as:** | | | | | |
| --- | --- | --- | --- | --- | --- |
|  | | Frequency | Percent | Valid Percent | Cumulative Percent |
| Valid | Allergy | 84 | 9.1 | 9.1 | 9.1 |
|  | Diabetes | 18 | 2.0 | 2.0 | 11.1 |
|  | Digistive disorder | 30 | 3.3 | 3.3 | 14.3 |
|  | Hypertension | 6 | .7 | .7 | 15.0 |
|  | No, I dont | 764 | 83.0 | 83.0 | 98.0 |
|  | Other | 18 | 2.0 | 2.0 | 100.0 |
|  | Total | 920 | 100.0 | 100.0 |  |

| **8. Did you avoid or limit your scheduled/routine visits to clinics/hospitals due to Covid-19** | | | | | |
| --- | --- | --- | --- | --- | --- |
|  | | Frequency | Percent | Valid Percent | Cumulative Percent |
| Valid | No | 150 | 16.3 | 16.3 | 16.3 |
|  | Yes | 770 | 83.7 | 83.7 | 100.0 |
|  | Total | 920 | 100.0 | 100.0 |  |

| **9. Did you prefer self-medication instead of visiting a doctor** | | | | | |
| --- | --- | --- | --- | --- | --- |
|  | | Frequency | Percent | Valid Percent | Cumulative Percent |
| Valid | No | 334 | 36.3 | 36.3 | 36.3 |
|  | Yes | 586 | 63.7 | 63.7 | 100.0 |
|  | Total | 920 | 100.0 | 100.0 |  |

| **10. Do you have any healthcare taker in the family as:** | | | | | |
| --- | --- | --- | --- | --- | --- |
|  | | Frequency | Percent | Valid Percent | Cumulative Percent |
| Valid | Doctor | 389 | 42.3 | 42.3 | 42.3 |
|  | No, I dont | 364 | 39.6 | 39.6 | 81.8 |
|  | Nurse | 36 | 3.9 | 3.9 | 85.8 |
|  | Pharmacist | 131 | 14.2 | 14.2 | 100.0 |
|  | Total | 920 | 100.0 | 100.0 |  |

| **11. Did you take advice from that family health caretaker** | | | | | |
| --- | --- | --- | --- | --- | --- |
|  | | Frequency | Percent | Valid Percent | Cumulative Percent |
| Valid | No | 306 | 33.3 | 33.3 | 33.3 |
|  | Yes | 614 | 66.7 | 66.7 | 100.0 |
|  | Total | 920 | 100.0 | 100.0 |  |

| **12. Your opinion for opting self-medication during Covid-19** | | | | | |
| --- | --- | --- | --- | --- | --- |
|  | | Frequency | Percent | Valid Percent | Cumulative Percent |
| Valid | Risky/Harmful | 263 | 28.6 | 28.6 | 28.6 |
|  | Safe/Helpful | 657 | 71.4 | 71.4 | 100.0 |
|  | Total | 920 | 100.0 | 100.0 |  |

| **13. Which of the following medications did you use for self- treatment** | | | | | |
| --- | --- | --- | --- | --- | --- |
|  | | Frequency | Percent | Valid Percent | Cumulative Percent |
| Valid | Anti-allergy | 162 | 17.6 | 17.6 | 17.6 |
|  | Antibiotics | 132 | 14.3 | 14.3 | 32.0 |
|  | Cough syrup | 132 | 14.3 | 14.3 | 46.3 |
|  | other | 132 | 14.3 | 14.3 | 60.7 |
|  | Painkillers | 362 | 39.3 | 39.3 | 100.0 |
|  | Total | 920 | 100.0 | 100.0 |  |

| **14. Did you get any side-effects after self-medication** | | | | | |
| --- | --- | --- | --- | --- | --- |
|  | | Frequency | Percent | Valid Percent | Cumulative Percent |
| Valid | No | 828 | 90.0 | 90.0 | 90.0 |
|  | Yes | 92 | 10.0 | 10.0 | 100.0 |
|  | Total | 920 | 100.0 | 100.0 |  |

| **15. If yes, then which of the following side effects did you feel after self-medication** | | | | | |
| --- | --- | --- | --- | --- | --- |
|  | | Frequency | Percent | Valid Percent | Cumulative Percent |
| Valid |  | 581 | 63.2 | 63.2 | 63.2 |
|  | Diarrhea | 12 | 1.3 | 1.3 | 64.5 |
|  | Dificulty in breathing | 12 | 1.3 | 1.3 | 65.8 |
|  | Drowsiness | 53 | 5.8 | 5.8 | 71.5 |
|  | Dry mouth | 47 | 5.1 | 5.1 | 76.6 |
|  | I don't have any side effects | 6 | .7 | .7 | 77.3 |
|  | NA | 6 | .7 | .7 | 77.9 |
|  | Nausea/Vomiting | 46 | 5.0 | 5.0 | 82.9 |
|  | No | 6 | .7 | .7 | 83.6 |
|  | No any side effects appears. | 6 | .7 | .7 | 84.2 |
|  | No one | 6 | .7 | .7 | 84.9 |
|  | No side effects | 17 | 1.8 | 1.8 | 86.7 |
|  | None | 30 | 3.3 | 3.3 | 90.0 |
|  | Nothing | 24 | 2.6 | 2.6 | 92.6 |
|  | Nothing happened | 6 | .7 | .7 | 93.3 |
|  | Stomach ache | 62 | 6.7 | 6.7 | 100.0 |
|  | Total | 920 | 100.0 | 100.0 |  |

| **16. If yes then how did you manage those side effects:** | | | | | |
| --- | --- | --- | --- | --- | --- |
|  | | Frequency | Percent | Valid Percent | Cumulative Percent |
| Valid |  | 548 | 59.6 | 59.6 | 59.6 |
|  | Drink plenty of water | 6 | .7 | .7 | 60.2 |
|  | Home remedy | 174 | 18.9 | 18.9 | 79.1 |
|  | I haven't seen side effects | 6 | .7 | .7 | 79.8 |
|  | I start taking lower dose of the medicine | 6 | .7 | .7 | 80.4 |
|  | Just kept myself hydrated | 6 | .7 | .7 | 81.1 |
|  | Limited use | 6 | .7 | .7 | 81.7 |
|  | Lots of tea | 6 | .7 | .7 | 82.4 |
|  | Nill | 6 | .7 | .7 | 83.0 |
|  | No any side effect appears | 6 | .7 | .7 | 83.7 |
|  | No side effects | 12 | 1.3 | 1.3 | 85.0 |
|  | None | 18 | 2.0 | 2.0 | 87.0 |
|  | Not applicable | 12 | 1.3 | 1.3 | 88.3 |
|  | Nothing | 18 | 2.0 | 2.0 | 90.2 |
|  | NS | 6 | .7 | .7 | 90.9 |
|  | Stopped taking medicines | 84 | 9.1 | 9.1 | 100.0 |
|  | Total | 920 | 100.0 | 100.0 |  |
